# Non-invasive mechanical ventilation and mortality in elderly immunocompromised patients hospitalized with pneumonia: a retrospective cohort study

**DOI:** 10.1186/1471-2466-14-7

**Published:** 2014-01-27

**Authors:** Christopher S Johnson, Christopher R Frei, Mark L Metersky, Antonio R Anzueto, Eric M Mortensen

**Affiliations:** 1 University of Texas Southwestern Medical Center, 5323 Harry Hines Blvd, Dallas, TX 75390, USA; 2 VA North Texas Health Care System, Dallas VA Medical Center, 4500 South Lancaster Rd, Dallas, TX 75216, USA; 3 South Texas Veterans Health Care System, 7400 Merton Minter Blvd, San Antonio, TX 8229, USA; 4 University of Texas Health Science Center at San Antonio, 7703 Floyd Curl Dr, San Antonio, TX 78229, USA; 5 University of Texas at Austin, 1 University Station A1900, Austin, TX 78712, USA; 6 University of Connecticut School of Medicine, 263 Farmington Ave, Farmington CT 06030, USA

**Keywords:** Mechanical ventilation, Mortality, Immunocompromised, Pneumonia

## Abstract

**Background:**

Mortality after pneumonia in immunocompromised patients is higher than for immunocompetent patients. The use of non-invasive mechanical ventilation for patients with severe pneumonia may provide beneficial outcomes while circumventing potential complications associated with invasive mechanical ventilation. The aim of our study was to determine if the use of non-invasive mechanical ventilation in elderly immunocompromised patients with pneumonia is associated with higher all-cause mortality.

**Methods:**

In this retrospective cohort study, data were obtained from the Department of Veterans Affairs administrative databases. We included veterans age ≥65 years who were immunocompromised and hospitalized due to pneumonia. Multilevel logistic regression analysis was used to determine the relationship between the use of invasive versus non-invasive mechanical ventilation and 30-day and 90-day mortality.

**Results:**

Of 1,946 patients in our cohort, 717 received non-invasive mechanical ventilation and 1,229 received invasive mechanical ventilation. There was no significant association between all-cause 30-day mortality and non-invasive versus invasive mechanical ventilation in our adjusted model (odds ratio (OR) 0.85, 95% confidence interval (CI) 0.66-1.10). However, those patients who received non-invasive mechanical ventilation had decreased 90-day mortality (OR 0.66, 95% CI 0.52-0.84). Additionally, receipt of guideline-concordant antibiotics in our immunocompromised cohort was significantly associated with decreased odds of 30-day mortality (OR 0.31, 95% CI 0.24-0.39) and 90-day mortality (OR 0.41, 95% CI 0.31-0.53).

**Conclusions:**

Our findings suggest that physicians should consider the use of non-invasive mechanical ventilation, when appropriate, for elderly immunocompromised patients hospitalized with pneumonia.

## Background

Pneumonia is the most common cause of death for all infectious diseases [1], and together, pneumonia and influenza comprise the eighth leading cause of death in the United States [2]. Much has been done to improve outcomes for patients with pneumonia, including the establishment of national practice guidelines for community-acquired pneumonia [3]. However, less information is available for immunocompromised patients. The immunocompromised are at an increased risk of developing pneumonia [4] and have a higher pneumonia mortality rate [5]. Further, the Infectious Diseases Society of America and the American Thoracic Society guidelines do not specifically address pneumonia amongst the immunocompromised [3].

Often, patients with severe pneumonia require endotracheal intubation and mechanical ventilation, which is associated with complications such as arrhythmia, infection, and other complications [6]. These complications may be more detrimental in immunocompromised patients. Non-invasive mechanical ventilation (NIV) is a method for delivering mechanical ventilation without employing the need for intubation [7]. Previous studies have evaluated the use of NIV in immunocompromised pneumonia patients [8-11]. Beneficial effects regarding the use of NIV have been demonstrated by several studies [9,12,13], while other studies have shown no benefits with its use [11,14].

The purpose of our study was to explore the association between all-cause mortality and NIV versus invasive mechanical ventilation for elderly patients (ages 65 and older) who were immunocompromised and admitted to the hospital with pneumonia. Our hypothesis is that all-cause mortality will be similar in patients receiving NIV and invasive mechanical ventilation.

## Methods

For this retrospective cohort study, data were obtained from the Department of Veterans Affairs (VA) Health Care System administrative databases, which contain clinical data from over 150 VA hospitals and 850 outpatient clinics. A prior paper provides the methods in more detail [15].

Patients were eligible to be included in this study if they:

a) Were hospitalized between October 1, 2001 and September 30, 2007.

b) Had a previously validated discharge diagnosis of pneumonia: either a primary diagnosis of pneumonia/influenza (ICD-9 codes 480.0-483.99 or 485–487) or a secondary discharge diagnosis of pneumonia with a primary diagnosis of respiratory failure (ICD-9 code 518.81) or sepsis (ICD-9 code 038.xx).

c) Were ≥65 years of age on admission.

d) Were immunocompromised: by their receipt of immunosuppressive medications, oral corticosteroids, acquired immune deficiency syndrome (AIDS) (ICD-9 code 42, 43, or 44), leukemia/multiple myeloma (ICD-9 code 203, 204, 205, 206, 207, or 208), or lymphoma (ICD-9: 200, 201, 202.0, 202.1, 202.2, 202.3, 202.5, 202.6, 202.7, 202.8, 202.9, 203.01, 203.80, 203.81, 238.6, 273.3, V10.71, V10.72, or V10.79)

e) Had at least one VA outpatient clinic visit in the year preceding admission.

f) Received at least one outpatient medication from a VA pharmacy within 90-days of admission.

g) Received either NIV or invasive mechanical ventilation during the hospitalization.

Patients were classified as taking an immunosuppressant medication if they received any of the following drugs within 90 days prior to admission: adalimumab/Humira, azathioprine, bleomycin, cisplatin, cyclophosphamide, cyclosporine, doxorubicin, daunorubicin, etanercept/Enbrel, fludarabine, infliximab/Remicade, methotrexate sodium, plicamycin/Mithracin, mitomycin, muromonab, mycophenolate/CellCept, natalizumab/Tysabri, prednisone, sirolimus, or tacrolimus. Also, patients were classified as taking oral corticosteroid therapy if they received any of the following medications within 90 days prior to admission: cortisone, dexamethasone, hydrocortisone, methylprednisolone, prednisolone, or prednisone.

### Invasive vs. non-invasive ventilation

A dichotomized variable indicating NIV (1) versus invasive mechanical ventilation (0) was created. NIV included both continuous positive airway pressure and intermittent positive pressure breathing, and was determined by the presence of ICD-9 codes 93.90 or 93.91. Invasive mechanical ventilation (intubation) status was determined by the presence of ICD-9 code 96.7x. Patients receiving both NIV and invasive mechanical ventilation during their index hospitalization were excluded from the analysis.

### Outcomes

Outcomes included all-cause 30-day and 90-day mortality. Mortality status was assessed using the VA Vital Status file.

### Covariates

Explanatory variables included age at admission, gender, drug counts within 90 days prior to admission (cardiovascular, diabetic, pulmonary, and inhaled corticosteroid), and number of primary clinical care and emergency department visits within 90 days prior to the admission. Indicator variables for smoking cessation status, alcohol abuse, drug abuse, hospital admission up to 90 days prior, race and ethnicity, marital status, VA priority group (a proxy for socioeconomic status), admission to the intensive care unit (ICU), nursing home residence prior admission, vasopressor use, and receipt of American Thoracic Society guideline-concordant antimicrobial therapy [3] were included, as well as cause/s of immunosuppression. VA priority groups are a way for the VA to focus limited funds to those veterans most in need. The highest group (priority group 1) must have at least a 50% service-connected disability. Priority groups 2 through 6 includes veterans with up to 40% service-connected disability, former prisoners of war, those awarded certain honors, veterans with lower incomes, and the catastrophically disabled. The lowest groups (priority groups 7 and 8) include veterans with relatively higher incomes who agree to pay copayments [16]. Additionally, prior ICD-9 codes were used to classify comorbidities using the previously validated Charlson-Deyo methodology (see Table 1) [17].

**Table 1:** Comparison of cohort by type of ventilatory support

**Variables**	**Non-invasive mechanical ventilation (N = 717)**	**Invasive mechanical ventilation (N = 1,229)**	***p*-value**
	**N (%)**	**N (%)**	
Age at admission, mean (SD)	75.8 (6.2)	75.3 (6.0)	0.07
Men	706 (98.5)	1216 (98.9)	0.36
Smoker	332 (46.3)	492 (40.0)	0.01
Alcohol abuse	30 (4.2)	64 (5.2)	0.31
Prior admission	203 (28.3)	409 (33.3)	0.02
Guideline concordant antibiotics	630 (87.9)	889 (72.3)	<0.01
Black	67 (9.3)	147 (12.0)	0.08
White	601 (83.8)	1014 (82.5)	0.46
Hispanic	24 (3.4)	93 (7.6)	<0.01
Married	393 (54.8)	633 (51.5)	0.16
VA priority group			0.97
1	137 (19.1)	229 (18.7)	
2–6	509 (71.0)	876 (71.3)	
7–8	71 (9.9)	123 (10.0)	
ICU admission	425 (59.3)	1124 (91.5)	<0.01
Myocardial infarction	45 (6.3)	116 (9.4)	0.02
Congestive heart failure	258 (36.0)	387 (31.5)	0.04
Peripheral vascular disease	108 (15.1)	194 (15.8)	0.67
Cerebrovascular disease	93 (13.0)	181 (14.7)	0.28
Dementia	13 (1.8)	34 (2.8)	0.19
Chronic obstructive pulmonary disease	590 (82.3)	821 (66.8)	<0.01
Rheumatologic disease	23 (3.2)	61 (5.0)	0.07
Peptic ulcer disease	24 (3.4)	44 (3.6)	0.79
Severe liver disease	5 (0.7)	17 (1.4)	0.19
Diabetes	257 (35.8)	411 (33.4)	0.28
Diabetes with complications	76 (10.6)	119 (9.7)	0.52
Chronic renal disease	85 (11.9)	174 (14.2)	0.15
Hemi/Paraplegia	10 (1.4)	17 (1.4)	0.98
Any prior malignancy	183 (25.5)	319 (26.0)	0.83
Metastatic solid tumor	29 (4.0)	56 (4.6)	0.59
Illicit drug abuse	9 (1.3)	15 (1.2)	0.95
Nursing home residence	10 (1.4)	7 (0.6)	0.06
Vasopressor use	50 (7.0)	499 (40.6)	<0.01
Cardiovascular drug count 90 days prior, mean (SD)	2.0 (1.6)	1.9 (1.6)	0.06
Diabetes drug count 90 days prior, mean (SD)	0.4 (0.8)	0.4 (0.7)	0.04
Inhaled corticosteroids drug count 90 days prior mean (SD)	0.75 (1.0)	0.62 (0.98)	0.01
Pulmonary drug count 90 days prior mean (SD)	2.4 (2.2)	2.0 (2.2)	<0.01
Number of primary care clinical visits 1 year prior, mean (SD)	5.0 (3.8)	5.0 (4.4)	0.88
Number of emergency department clinic visits 1 year prior, mean (SD)	1.3 (3.6)	1.4 (2.8)	0.52
Reason for immunosuppression			
Taking immunosuppressive drugs	611 (85.2)	961 (78.2)	<0.01
Leukemia/lymphoma/multiple myeloma	42 (5.9)	121 (9.9)	<0.01
Taking oral corticosteroids	362 (50.5)	649 (52.8)	0.32
AIDS	3 (0.4)	3 (0.2)	0.68

### Statistical analysis

Demographic, utilization, and comorbidity differences between NIV and invasive mechanical ventilation groups were assessed using the Student’s t-test for continuous variables and the χ^2^ test and Fisher’s exact test for categorical variables. A multilevel logistic regression analysis was performed to determine factors associated with the receipt of NIV (1) versus invasive (0) mechanical ventilation.

Univariate logistic regression analyses were performed to evaluate the associations between mortality (30-day or 90-day) and the dichotomized mechanical ventilation exposure, as well as other prognostic variables. Multilevel logistic regression analyses were performed with the dichotomized mechanical ventilation exposure (non-invasive vs. invasive) as the independent variable. Other covariates included the model are listed in Table 1. Patients were considered grouped within VA facilities. Interaction between smoking status and number of inhaled corticosteroids was evaluated, as well.

In addition, we performed sub-analyses using only patients admitted to the ICU and only patients admitted to the ICU who were also taking vasopressors to account for possible differences in acute severity of illness between groups. Vasopressor use for hypovolemia is classified as a major criteria for severe community acquired pneumonia according to the Infectious Diseases Society of America and the American Thoracic Society guidelines [3], and so those patients receiving vasopressor medications during their stay should be more severely ill. Also, it has been shown that the increased use of non-invasive ventilation, when used on patients with acute exacerbations of COPD, has resulted in significant reductions in mortality [18]. Therefore, to minimize the conceivable effect seen in this group of patients, we performed a sub-analysis of patients without COPD. Further, we performed propensity score matching, based on the same covariates in the logistic regressions models, to adjust for known confounders. This resulted in matching 422 pairs of patients with no statistically significant differences in any of the variables listed in Table 1. Lastly, we performed multilevel logistic regression analyses incorporating time (fiscal year of admission) into the model on the full cohort and sub-cohorts, as well as conditional logistic regression using a propensity-matched cohort created with time incorporated into the model to control for any possible effect of time on mortality. A Kaplan-Meier curve was generated to assess 90-day survival between groups. The log-rank test was utilized to assess differences in survival.

Statistical significance was found if *p <* 0.05. All analyses were performed using STATA 12 (StataCorp, College Station, TX).

## Results

We identified 1,946 patients who met the inclusion and exclusion criteria. By cause of immunosuppression, 1,572 (81%) had received immunosuppressive medications, 1,011 (52%) received oral corticosteroids, 6 (0.3%) had acquired immunodeficiency syndrome, and 163 (8%) had a history of leukemia, lymphoma, and/or multiple myeloma. Of this cohort, 717 (37%) received NIV and 1,229 (63%) received invasive mechanical ventilation. Baseline differences between these two groups are displayed in Table 1.

### Predictors of non-invasive mechanical ventilation

There were many variables that were significantly associated with NIV in the univariate analysis. As displayed in Table 1, these included smoking cessation status, receipt of guideline concordant antibiotics, congestive heart failure, chronic obstructive pulmonary disease, vasopressor use, elevated diabetic, inhaled corticosteroid and pulmonary drug counts, and receipt of immunosuppressive medications.

Table 2 displays predictors of receipt of NIV in the multilevel regression model. Receipt of guideline concordant antibiotics, congestive heart failure, chronic obstructive pulmonary disease, and increased inhaled corticosteroid drug count within 90 days prior to admission were all significantly associated with receipt of NIV. ICU admission and vasopressor use were both significantly associated with a decreased likelihood of receiving NIV.

**Table 2:** Results of multivariable regression model for outcome of non-invasive mechanical ventilation

**Variables**	**OR**	**95% CI**		***p*-Value**
Age at admission	1.02	1.00	1.04	0.12
Men	0.52	0.18	1.45	0.21
Smoke	1.34	0.98	1.83	0.07
Alcohol abuse	0.80	0.44	1.46	0.46
Prior admission	0.79	0.59	1.06	0.12
Guideline concordant antibiotics	1.59	1.14	2.22	0.01
Black	0.71	0.37	1.36	0.30
White	0.86	0.51	1.47	0.59
Hispanic	0.96	0.43	2.11	0.91
Married	1.10	0.85	1.42	0.47
VA priority groups 2–6	0.95	0.69	1.31	0.76
VA priority groups 7–8	1.11	0.68	1.81	0.68
ICU	0.15	0.10	0.20	<0.01
Reason for immunosuppression				
Taking immunosuppressive drugs	1.11	0.76	1.61	0.61
Leukemia/lymphoma/multiple myeloma	0.86	0.52	1.41	0.55
Taking oral corticosteroids	1.05	0.79	1.39	0.73
AIDS	4.78	0.49	46.76	0.18
Myocardial infarction	0.64	0.40	1.03	0.06
Congestive heart failure	1.36	1.03	1.80	0.03
Peripheral vascular disease	0.83	0.59	1.18	0.30
Cerebrovascular disease	0.93	0.64	1.34	0.70
Dementia	0.89	0.38	2.09	0.79
Chronic obstructive pulmonary disease	2.01	1.43	2.81	<0.01
Rheumatologic disease	0.53	0.27	1.03	0.06
Peptic ulcer disease	0.88	0.44	1.74	0.71
Severe liver disease	0.60	0.18	1.96	0.39
Diabetes	0.96	0.66	1.38	0.81
Diabetes with complications	0.98	0.61	1.56	0.92
Chronic renal disease	0.97	0.66	1.42	0.87
Hemi/Paraplegia	1.30	0.45	3.78	0.63
Any prior malignancy	1.19	0.88	1.61	0.26
Metastatic solid tumor	1.62	0.87	3.03	0.13
Illicit drug abuse	0.92	0.31	2.75	0.88
Nursing home residence	1.13	0.31	4.17	0.85
Vasopressor use	0.15	0.10	0.21	<0.01
Cardiovascular drug count 90d prior	1.03	0.95	1.13	0.44
Diabetes drug count 90d prior	1.26	0.99	1.60	0.06
Inhaled corticosteroid drug count 90d prior	1.19	1.01	1.41	0.04
Pulmonary drug count 90 days prior	0.98	0.92	1.05	0.56
# of primary care clinical visits 1 year prior	1.00	0.96	1.03	0.80
# of emergency department clinic visits 1 year prior	1.01	0.96	1.06	0.70

### 30-day mortality

Results of the multilevel logistic regression models are shown in Tables 3 and 4. NIV was not significantly associated with 30-day mortality (OR 0.85, 95% CI 0.66-1.10). This effect was also not significant in any of our sub-analyses, as shown in Table 5, nor was it significant in our models incorporating fiscal year of admission. However, higher age at admission (OR 1.04, 95% CI 1.03-1.06), any prior hospital admission (OR 1.41, 95% CI 1.10-1.80), VA priority groups 2 through 6 (OR 1.57, 95% CI 1.17-2.11) and 7 through 8 (OR 2.06, 95% CI 1.34-3.17), any prior malignancy (OR 1.32, 95% CI 1.02-1.71), metastatic solid tumor (OR 2.49, 95% CI 1.45-4.30), and vasopressor use (OR 1.75, 95% CI 1.37-2.24) were significantly associated with increased mortality. Receipt of guideline concordant antibiotics (OR 0.31, 95% CI 0.24-0.39), black race (OR 0.53, 95% CI 0.30-0.93) and increasing number of cardiovascular medications (OR 0.92, 95% CI 0.85-0.99) were associated with decreased mortality. There was no significant interaction between smoking status and number of inhaled corticosteroids. When examining the relationship between the cause of immunosuppression and 30-day mortality, leukemia, lymphoma, and/or multiple myeloma (OR 1.96, 95% CI 1.33-2.89) and receipt of oral corticosteroids (OR 1.89, 95% CI 1.48-2.42) were associated with increased mortality.

**Table 3:** Results of multivariable regression model for outcome of 30-day mortality

**Variables**	**OR**	**95% CI**		***p*-Value**
Non-invasive mechanical ventilation	0.85	0.66	1.10	0.23
Age at admission	1.04	1.03	1.06	<0.01
Men	0.43	0.17	1.09	0.08
Smoke	0.87	0.66	1.14	0.30
Alcohol abuse	0.60	0.34	1.05	0.08
Prior admission	1.41	1.10	1.80	0.01
Guideline concordant antibiotics	0.31	0.24	0.39	<0.01
Black	0.53	0.30	0.93	0.03
White	0.89	0.57	1.41	0.63
Hispanic	0.70	0.43	1.13	0.14
Married	0.90	0.72	1.12	0.34
VA priority groups 2–6	1.57	1.17	2.11	<0.01
VA priority groups 7–8	2.06	1.34	3.17	<0.01
ICU	1.13	0.84	1.53	0.41
Reason for immunosuppression				
Taking immunosuppressive drugs	0.74	0.55	0.99	0.05
Leukemia/lymphoma/multiple myeloma	1.96	1.33	2.89	<0.01
Taking oral corticosteroids	1.89	1.48	2.42	<0.01
AIDS	1.38	0.16	11.59	0.77
Myocardial infarction	0.96	0.65	1.44	0.86
Congestive heart failure	1.00	0.78	1.28	1.00
Peripheral vascular disease	1.23	0.91	1.65	0.18
Cerebrovascular disease	1.03	0.75	1.41	0.87
Dementia	1.12	0.56	2.22	0.75
Chronic obstructive pulmonary disease	0.88	0.67	1.17	0.38
Rheumatologic disease	1.16	0.70	1.91	0.58
Peptic ulcer disease	1.02	0.57	1.81	0.96
Severe liver disease	2.11	0.77	5.78	0.15
Diabetes	0.87	0.63	1.20	0.40
Diabetes with complications	0.92	0.61	1.37	0.67
Chronic renal disease	1.08	0.78	1.50	0.64
Hemi/Paraplegia	0.53	0.21	1.35	0.19
Any prior malignancy	1.32	1.02	1.71	0.03
Metastatic solid tumor	2.49	1.45	4.30	<0.01
Illicit drug abuse	1.53	0.61	3.87	0.37
Nursing home residence	0.55	0.16	1.91	0.35
Vasopressor use	1.75	1.37	2.24	<0.01
Cardiovascular drug count 90d prior	0.92	0.85	0.99	0.04
Diabetes drug count 90d prior	1.03	0.84	1.28	0.76
Inhaled corticosteroid drug count 90d prior	0.99	0.85	1.14	0.85
Pulmonary drug count 90 days prior	0.99	0.93	1.05	0.80
# of primary care clinical visits 1 year prior	1.01	0.99	1.04	0.31
# of ED clinic visits 1 year prior	1.02	0.98	1.05	0.36

**Table 4:** Results of multivariable regression model for outcome of 90-day mortality

**Variables**	**OR**	**95% CI**		***p*-Value**
Non-invasive mechanical ventilation	0.66	0.52	0.84	<0.01
Age at admission	1.06	1.04	1.08	<0.01
Men	0.70	0.28	1.75	0.44
Smoke	0.73	0.56	0.94	0.02
Alcohol abuse	0.71	0.43	1.18	0.18
Prior admission	1.56	1.23	1.98	<0.01
Guideline concordant antibiotics	0.41	0.31	0.53	<0.01
Black	0.37	0.21	0.63	<0.01
White	0.60	0.39	0.93	0.02
Hispanic	0.88	0.54	1.44	0.62
Married	0.89	0.72	1.10	0.29
VA priority groups 2–6	1.40	1.07	1.83	0.02
VA priority groups 7–8	1.47	0.97	2.21	0.07
ICU	1.25	0.95	1.66	0.11
Reason for immunosuppression				
Taking immunosuppressive drugs	0.83	0.62	1.13	0.24
Leukemia/lymphoma/multiple myeloma	1.75	1.17	2.60	0.01
Taking oral corticosteroids	1.67	1.32	2.10	<0.01
AIDS	2.63	0.35	20.07	0.35
Myocardial infarction	1.09	0.74	1.59	0.67
Congestive heart failure	1.09	0.86	1.38	0.47
Peripheral vascular disease	1.09	0.82	1.45	0.54
Cerebrovascular disease	1.03	0.76	1.39	0.84
Dementia	1.08	0.55	2.13	0.82
Chronic obstructive pulmonary disease	1.01	0.78	1.33	0.92
Rheumatologic disease	0.65	0.40	1.08	0.10
Peptic ulcer disease	1.18	0.68	2.05	0.56
Severe liver disease	3.87	1.32	11.32	0.01
Diabetes	0.90	0.66	1.22	0.50
Diabetes with complications	0.83	0.57	1.22	0.34
Chronic renal disease	1.16	0.84	1.59	0.36
Hemi/Paraplegia	0.64	0.26	1.57	0.33
Any prior malignancy	1.27	0.98	1.63	0.07
Metastatic solid tumor	3.44	1.85	6.37	<0.01
Illicit drug abuse	1.43	0.56	3.64	0.45
Nursing home residence	0.30	0.09	1.05	0.06
Vasopressor use	2.04	1.59	2.60	<0.01
Cardiovascular drug count 90d prior	0.95	0.88	1.02	0.16
Diabetes drug count 90d prior	1.15	0.94	1.40	0.17
Inhaled corticosteroid drug count 90d prior	1.00	0.87	1.15	0.97
Pulmonary drug count 90 days prior	0.98	0.93	1.04	0.60
# of primary care clinical visits 1 year prior	1.02	0.99	1.05	0.20
# of ED clinic visits 1 year prior	1.01	0.98	1.05	0.55

**Table 5:** Effect of non-invasive mechanical ventilation on 30-day mortality and 90-day mortality

	**Full cohort multivariable model (n = 1925)**	**ICU Sub-cohort multivariable model (n = 1531)**	**ICU + Vasopressor use sub-cohort multivariable model (n = 514)**	**Propensity-matched cohort (n = 844)**	**Sub-cohort without COPD multivariable model (n = 531)**
	**OR (95% CI)**	**OR (95% CI)**	**OR (95% CI)**	**OR (95% CI)**	**OR (95% CI)**
**30-day mortality**	0.85 (0.66, 1.10)	1.05 (0.78, 1.41)	0.68 (0.31, 1.49)	0.97 (0.72, 1.30)	0.87 (0.50-1.54)
**90-day mortality**	0.66 (0.52, 0.84)^*^	0.73 (0.55, 0.96)^*^	0.46 (0.22, 0.97)^*^	0.74 (0.56, 0.98)^*^	0.74 (0.44-1.26)

*Indicates p <0.05.

### 90-day mortality

**Figure 1:**
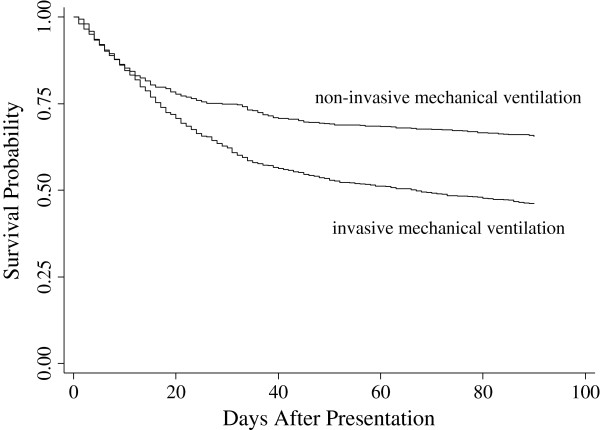
**Kaplan-Meier plot of 90-day survival.** Patients undergoing non-invasive mechanical ventilation had significantly higher survival than did invasive ventilation patients (*p <* 0.001 by the log-rank test).

Figure 1 displays the Kaplan-Meier 90-day survival curves for the NIV and invasive mechanical ventilation groups. We found that NIV patients had significantly higher survival than did invasive ventilation patients (*p* <0.001).

As shown in Table 4, NIV was associated with decreased mortality in the multilevel logistic regression analysis for 90-day mortality (OR 0.66, 95% CI 0.52-0.84). This effect remained significant in the ICU, ICU with vasopressor use, and propensity-matched sub-group analyses but not in the sub-analysis of patients without comorbid COPD, as shown in Table 5. Additionally, there were negligible differences when fiscal year of admission was incorporated into the model. Smoking cessation (OR 0.73, 95% CI 0.56-0.94), receipt of guideline concordant antibiotics (OR 0.41, 95% CI 0.31-0.53), black race (OR 0.37, 95% CI 0.21-0.63), and white race (OR 0.60, 95% CI 0.39-0.93) were also significantly associated with decreased risk of 90-day mortality. Conversely, higher age at admission (OR 1.06, 95% CI 1.04-1.08), prior hospital admission (OR 1.56, 95% CI 1.23-1.98), being in VA priority groups 2 through 6 (OR 1.40, 95% CI 1.07-1.83), severe liver disease (OR 3.87, 95% CI 1.32-11.32), metastatic solid tumor (OR 3.44, 95% CI 1.85-6.37), and vasopressor use (OR 2.04, 95% CI 1.59-2.60) were significantly associated with increased 90-day mortality. There was no significant interaction between smoking status and number of inhaled corticosteroids. In examining the association between cause of immunosuppression and 90-day mortality, we found leukemia, lymphoma, and/or multiple myeloma (OR 1.75, 95% CI 1.17-2.60) and receipt of oral corticosteroids within 90 days prior to index admission (OR 1.67, 95% CI 1.32-2.10) to be associated with increased mortality.

## Discussion

We found the use of NIV in elderly hospitalized immunocompromised pneumonia patients to be associated with decreased mortality at 90-days, but not at 30-days, after adjusting for potential confounders. Additionally, we observed that the receipt of guideline-concordant antibiotics to be associated with decreased odds of mortality, at both 30- and 90-days. These data suggest that physicians should consider the use of NIV, when appropriate, for elderly immunocompromised patients hospitalized with pneumonia. At minimum, patients receiving NIV fared no worse than similar patients receiving invasive ventilation.

Though previous studies have evaluated NIV in immunocompromised patients, many have not specifically examined mortality rates of NIV versus invasive mechanical ventilation for immunocompromised pneumonia patients. Similar studies have investigated and found a beneficial association between NIV use and survival in patients with hematological malignancies [19,20]. Our study too found similar beneficial effects of NIV on mortality, even while specifically restricting to pneumonia patients and including other forms of immunosuppression. Another prior study of patients with severe acute hypoxemic respiratory failure found that patients receiving NIV had a significantly decreased risk of 90-day mortality when compared to patients receiving high-concentration oxygen. However, this study did not strictly evaluate immunocompromised patients, and it did not compare 90-day mortality between patients receiving NIV with those receiving invasive ventilation [21]. A study by Hilbert et al. [9] evaluated NIV for immunocompromised patients with pulmonary infiltrates, fever, and early stages of hypoxemic acute respiratory failure. The authors found improvements in in-hospital mortality for patients receiving NIV, but the patients in this study were assessed prior to the need for intubation, and so had not reached a critical state. Further, the use of NIV was only compared to the use of standard treatment with supplemental oxygen and no ventilatory support. Lastly, a study by Razlaf and colleagues [22] investigated NIV failure in immunocompromised patients. This study also demonstrated that NIV in immunocompromised patients resulted in weaning to spontaneous breathing and eventual hospital discharge in almost half of study subjects. Though mortality was evaluated, this study proved that severity of illness at ICU admission was a significant predictor for NIV failure, which may explain why some patients who are more critically ill receive invasive ventilation instead of NIV.

Our study specifically examines all-cause mortality and its association with the use of NIV and invasive mechanical ventilation for elderly immunocompromised patients with pneumonia. We found a positive association between the use of NIV and mortality, particularly when we investigated all-cause 90-day mortality. To our knowledge, this has not been shown in previous studies. In our univariate analyses, the use of NIV was associated with lower 30-day and 90-day mortality. After adjusting for demographic, utilization, and comorbid factors, as well as underlying cause of immunosuppression, NIV was only associated with lower mortality after 90 days. It has previously been shown that pneumonia plays a primary role in death within 30 days of hospitalization for patients diagnosed with community-acquired pneumonia. Likewise, comorbid conditions are more likely to play a prominent role in death within 90 days [23]. The same may be true for immunocompromised patients with pneumonia. Accordingly, the same comorbidities that contribute to the need for invasive mechanical ventilation may also be contributing to the higher mortality after 30-days we found for patients receiving invasive mechanical ventilation. However, we repeated our analyses only in patients admitted to the ICU and only in patients admitted to the ICU who were also taking vasopressors, in an attempt to equalize possible differences in severity of illness between the groups. Likewise, we repeated our analyses on patients without comorbid COPD. Even still, those patients receiving NIV did no worse than those receiving invasive mechanical ventilation. We also used propensity score matching to create a cohort of patients with balanced baseline covariates between groups. The effect of NIV remained significant for 90-day mortality and insignificant for 30-day mortality in all of these sub-cohort analyses, as shown in Table 5. Our survival analysis indicated a significantly higher 90-day survival probability for those who received NIV compared to those who did not. The Kaplan-Meier curve shows that the risk of mortality is comparable between the two groups for about the first 10 days, after which the invasive mechanical ventilation group appears to have a sharper drop in survival. This may suggest invasive mechanical ventilation could lead to complications contributing to elevated mortality over a longer period of time.

Aside from the use of NIV, we also identified other interesting variables significantly associated with 30-day and 90-day mortality. One significant finding in our study related to the use of antibiotics concordant with the American Thoracic Society / Infectious Diseases Society of America community-acquired pneumonia guidelines. Though the guidelines put forth by these societies do not address immunocompromised patients, we found these recommendations to be highly beneficial for immunocompromised patients for both 30-day and 90-day mortality.

Further, we examined the underlying cause of immunosuppression and its relationship to our outcomes. We found receipt of immunosuppressive medications to be associated with decreased 30-day and 90-day mortality. This association may be explained by the concept that patients receiving immunosuppressive medications are under closer watch by their physicians. As such, these patients may receive more timely care than others.

Our study has limitations. First, we did not have clinical information such as physical exam, laboratory, or radiographic data. Consequently, we could not account for patients who may have responded more favorably to NIV, such as patients with hypercapnic respiratory failure. Second, we did not include specific measures of severity in our analysis, such as the Pneumonia Severity Index or CURB-65, as we were missing several necessary variables. However, we did adjust for several demographic elements and comorbid conditions, which are included in these measures. Likewise, we performed sub-group analyses using only ICU patients and only ICU patients receiving vasopressors in an effort to make non-invasive and invasive mechanical ventilation patients comparable in level of severity and reduce selection bias. Further, we could not account for contraindications for use of NIV, such as upper gastrointestinal obstruction, gastrointestinal bleeding, or recent upper gastrointestinal, facial, or upper airway obstruction. As such, patients with these conditions would not be candidates for NIV, due to the mere fact that they had these conditions. Additionally, it is possible that patients diagnosed with obstructive sleep apnea and treated with continuous positive airway pressure may have skewed the results. Lastly, data regarding NIV failure was unavailable to us. It has previously been shown that patients failing NIV have an overall increased mortality, even greater than patients receiving elective invasive ventilation [19]. However, patients receiving both invasive and non-invasive ventilation during their index hospitalization were removed from our analyses, thus eliminating this possible bias.

## Conclusions

Our study reveals significant differences in mortality between those elderly immunocompromised patients with pneumonia who received NIV versus those who received invasive mechanical ventilation. Specifically, we found a beneficial association between the use of NIV and mortality. Additionally, the use of guideline-concordant antibiotics in the immunocompromised with pneumonia was significantly associated with lower 30-day and 90-day mortality. Though our study is observational in nature, overall our results suggest the use of NIV in this elderly patient population is associated with decreased mortality as compared to invasive mechanical ventilation. Therefore, physicians should consider the use of NIV, when appropriate, for immunocompromised patients hospitalized with pneumonia. However, additional studies, especially randomized controlled trials, are needed to confirm our findings.

## Abbreviations

OR: Odds ratio; CI: Confidence interval; AIDS: Acquired immune deficiency syndrome; ICU: Intensive care unit; NIV: Non-invasive mechanical ventilation; VA: Department of Veterans affairs.

## Competing interests

No competing interests exist for Christopher Johnson, Christopher Frei, Mark Metersky, Antonio Anzueto, or Eric Mortensen.

## Authors’ contributions

CJ was responsible for study concept and design, analysis and interpretation of data, drafting of the manuscript; CF was responsible for interpretation of the data, drafting of the manuscript, and critical revision of manuscript for important intellectual content; MM was responsible for interpretation of the data, drafting of the manuscript, and critical revision of manuscript for important intellectual content; AA was responsible for interpretation of the data, drafting of the manuscript, and critical revision of manuscript for important intellectual content; EM was responsible for study concept and design, interpretation of the data, drafting of the manuscript, and critical revision of manuscript for important intellectual content. All authors read and approved the final manuscript.

## Pre-publication history

The pre-publication history for this paper can be accessed here:

http://www.biomedcentral.com/1471-2466/14/7/prepub
